# Socially priming dogs in an overimitation task

**DOI:** 10.3389/fpsyg.2023.1063132

**Published:** 2023-02-17

**Authors:** Louise Mackie, Ludwig Huber

**Affiliations:** Comparative Cognition, Messerli Research Institute, University of Veterinary Medicine Vienna, Vienna, Austria

**Keywords:** overimitation, priming, dog behavior, social learning, comparative psychology

## Abstract

Overimitation — the copying of another’s unnecessary or irrelevant actions toward a goal — is largely considered to be uniquely human. Recent studies, however, have found evidence of this behavior in dogs. Humans seem to overimitate more or less depending on social factors, such as the cultural origin of the demonstrator. Like humans, dogs may have social motivations behind their overimitation, since they have been shown to copy irrelevant actions more from their caregivers than from strangers. By using priming methodology, this study aimed to investigate whether dogs’ overimitation can be facilitated *via* the experimental manipulation of their attachment-based motivations. To test this, we invited caregivers to demonstrate goal-irrelevant and relevant actions to their dog, following either a dog-caregiver relationship prime, a dog-caregiver attention prime, or no prime. Our results showed no significant main effect of priming on copying behavior for either relevant or irrelevant actions, but we found a trend that unprimed dogs copied the least actions overall. Additionally, dogs copied their caregiver’s relevant actions more often and more faithfully as the number of trials increased. Our final finding was that dogs were much more likely to copy irrelevant actions after (rather than before) already achieving the goal. This study discusses the social motivations behind dog imitative behavior, and has potential methodological implications regarding the influence of priming on dog behavioral studies.

## 1. Introduction

We, as humans, watch and imitate other people when encountering uncertain or novel situations, such as having that first cup of tea in the breakroom when starting a new job. We also often copy other people’s goal-irrelevant actions, like pointing out our pinky finger while holding said cup of tea. This phenomenon, called *overimitation* (reviewed in Legare and Nielsen, 2015; Hoehl et al., 2019; Whiten, 2019), is prevalent all the way from childhood to adulthood, and is said to be responsible for the transmission of traditional and cultural behaviors in our societies (Flynn and Smith, 2012). Whether or not humans choose to copy goal-irrelevant actions, as well as goal-relevant ones, at least partially depends on social factors. Five and six-year-old children, for example, have been shown to display higher levels of overimitation when the action-demonstrator is of the same cultural background (Krieger et al., 2020), perceived as a prosocial person (Schleihauf and Hoehl, 2021), or when the demonstrator is in-person rather than on a video-recording (McGuigan et al., 2007). It seems that the motivation to copy irrelevant actions is driven by a desire to affiliate with or “be like” the demonstrator. In other words, *whom* an individual is copying matters.

Despite an extensive body of research on overimitation in humans, there are very few studies investigating it in other animals. The available studies on non-human primates have reported a lack of irrelevant-action copying, inferring human uniqueness [e.g., chimpanzees (*Pan troglodytes*); Horner and Whiten, 2005; bonobos (*Pan paniscus*); Clay and Tennie, 2018; orangutans (*Pongo pygmaeus*); Nielsen and Susianto, 2010], but these studies have only used human demonstrators. Dogs (*Canis familiaris*), compared to non-human primates, share a special relationship with humans due to their domestication process (Range and Virányi, 2015) and, over thousands of years, have become adapted to living within human societies (Miklósi et al., 2004). They can form close attachments with people, respond to our ostensive gestures, and even be trained to help us by detecting seizures yet to occur (Brown and Goldstein, 2011; Catala et al., 2019). Additionally, dogs have been reported to not only imitate human actions, such as jumping in “do as I do” tasks (Fugazza and Miklósi, 2014), but also to overimitate human actions by touching functionless dots on a wall, especially when the human demonstrator is the dog’s caregiver rather than a stranger (Huber et al., 2018, 2020; but see, Johnston et al., 2017). Huber et al. (2022) explored the quality of the dog-caregiver relationship in cases of overimitation, finding that the dogs who accurately copied irrelevant actions from their caregiver also showed affiliative behaviors toward them, such as gazing and synchronization. Collectively, these studies suggest that, like human children, dogs may overimitate because of social motivations elicited by the demonstrator. Experimental manipulation of these motivations could help pinpoint their *causal* influence on imitation behavior, which is an important next step in the literature on overimitation in dogs.

An individual’s social motivations are not fixed, but influenceable by the surrounding context or internal feelings. Priming methodology is a way to direct one’s motivation, by exposing an individual to certain stimuli prior to an experimental task. Priming relies on timing and order, so the immediate effects are the most influential. Human children are often subjects of priming studies in which they are presented with pictures, games, or videos to induce certain feelings before participating in social tasks. For example, after experiencing third-party social exclusion (ostracism), children more faithfully copied an experimenter who demonstrated to them how to turn on a light box (Over and Carpenter, 2009). The primed children in this study copied more irrelevant actions, such as the rolling of a tool in one’s hand, indicating that their motivation to affiliate with the demonstrator was temporarily enhanced by their feelings of ostracism. Priming has also been used to manipulate feelings of attachment security in 6 and 7-year-old children; by subliminally flashing images of a mother–child pair, Stupica et al. (2019) found that priming attachment changed children’s physiological responses to threatening pictures.

The current study aimed to investigate whether priming (focused on the dog-caregiver bond) can influence dogs’ social motivation and their copying behavior. Since priming has successfully influenced children’s affiliative attitudes, we hypothesized that priming would analogously facilitate a dog’s motivation to copy their caregiver. In addition, since the previous work on overimitation in dogs suggested a role of the dog-caregiver bond, we chose primes that emphasized this relationship to test how it may causally influence dog behavior in Huber et al.’s overimitation task (2018). We separated copying behavior into relevant and irrelevant action-copying, as priming could affect motivations for these differently (the goal involves a food reward). We also chose to use one order of actions in the caregiver’s demonstration (an irrelevant-then-relevant action-sequence), as the reverse order had been explored in the original studies with no effect (Huber et al., 2018, 2020).

We had three between-subject conditions of dogs; two conditions experienced separate primes before the overimitation task, and one condition completed the overimitation task without any prime. Dogs in *the relationship prime* experienced a prime that was intended to activate the dog’s attachment-system with their caregiver. Attachment behavior may be activated by a fear-inducing event, and a common method to prompt this behavior is to use the Threatening Stranger (TS) procedure (Vas et al., 2005; Solomon et al., 2019), in which a stranger slowly approaches a dog in a mildly threatening manner. This method can also help assess to what extent a dog can use its caregiver as a physical point of safety — a safe-haven. Thus, we used the TS procedure as our relationship prime, and, as a means to investigate the attachment quality of each dog in relation to overimitation. We predicted that those dogs who overimitate would have a stronger safe-haven response than those who do not, since this response can indicate relationship quality (Cimarelli et al., 2016), and since the top overimitators in Huber et al.’s (2022) study had good relationship quality with their caregiver. Dogs in *the attention prime* participated in a prime that was designed to enhance caregiver-directed attention. This prime was a cup-game, akin to a warm-up task that can increase one’s attention. For example, imitation studies for human children typically have warm-up games before their imitation tasks, like a game of marbles (Schleihauf and Hoehl, 2021) or drawing pictures (Over and Carpenter, 2009). Warm-ups such as these help to create a context of engagement and an attentive mood, acting like a prime on children’s performance for the main task of the study. Sassenberg et al. (2017) intentionally explored the benefits of attentional priming, and found that when adults completed a pre-task questionnaire on creativity they then showed amplified originality in a brainstorming task. The task-performance of dogs may also improve from this type of priming. So, we predicted that by experiencing a pre-task cup-game (from Huber et al., 2018) with their caregivers, dogs would have increased attention and performance in the overimitation task.

## 2. Materials and methods

### 2.1. Ethical statement

This study and its procedures were discussed and approved by the ethics committee of the University of Veterinary Medicine Vienna, in accordance with the good scientific practice and national legislation guidelines (reference: ETK-182/11/2021). Caregiver participation was voluntary with written consent, and their dogs experienced non-invasive behavioral tests for food rewards. Handling was done in a friendly manner, and caregivers remained in proximity to their dogs for the duration of the session at the Clever Dog Lab.

### 2.2. Subjects

Seventy-one dogs were tested with their caregivers at the Clever Dog Lab of the Veterinary Medicine University of Vienna. There were 58 dogs in the final sample (27 males, 31 females) after dogs were excluded for substantial distraction or because their caregiver attempted to assist them during trials. Nine (out of 36) caregivers brought more than one dog for testing. The waiting dogs were placed in an unoccupied testing room with a bowl of water and an available researcher in case of need. Recruited dogs were asked to be food motivated, be between one and 12-years-old (*M*_age_ = 4.64 years), and to have never participated in an overimitation study at the Clever Dog Lab to maintain task-novelty. Caregivers were always asked if their dog could eat sausage, and if not, they were asked to bring appropriate food so that the reward was always familiar to the dog. There were three between-subject conditions (1, *N* = 19, 2; *N* = 19, 3; *N* = 20), where dogs were assigned based on age, sex, and breed for counterbalancing. Although the conditions were closely balanced, the total sample had a slight overrepresentation of Border Collies (*N* = 9) and Mixed dogs (*N* = 8), while other breeds were represented by five or less. There was also an overrepresentation of 1 and 2-year-old dogs (*N* = 20). The details of individual dogs (including their behavioral score, condition, age, sex, breed, training history, personality ratings, and whether they had prior experience of the Clever Dog Lab) are provided in the datasheet of the Supplementary material. Most dogs had been at least once to the Clever Dog Lab for previous testing (50/58 dogs).

There was one female experimenter (LM) for this study, who was unfamiliar with all of the participating dogs and caregivers.

### 2.3. Design and procedure

**Figure 1:**
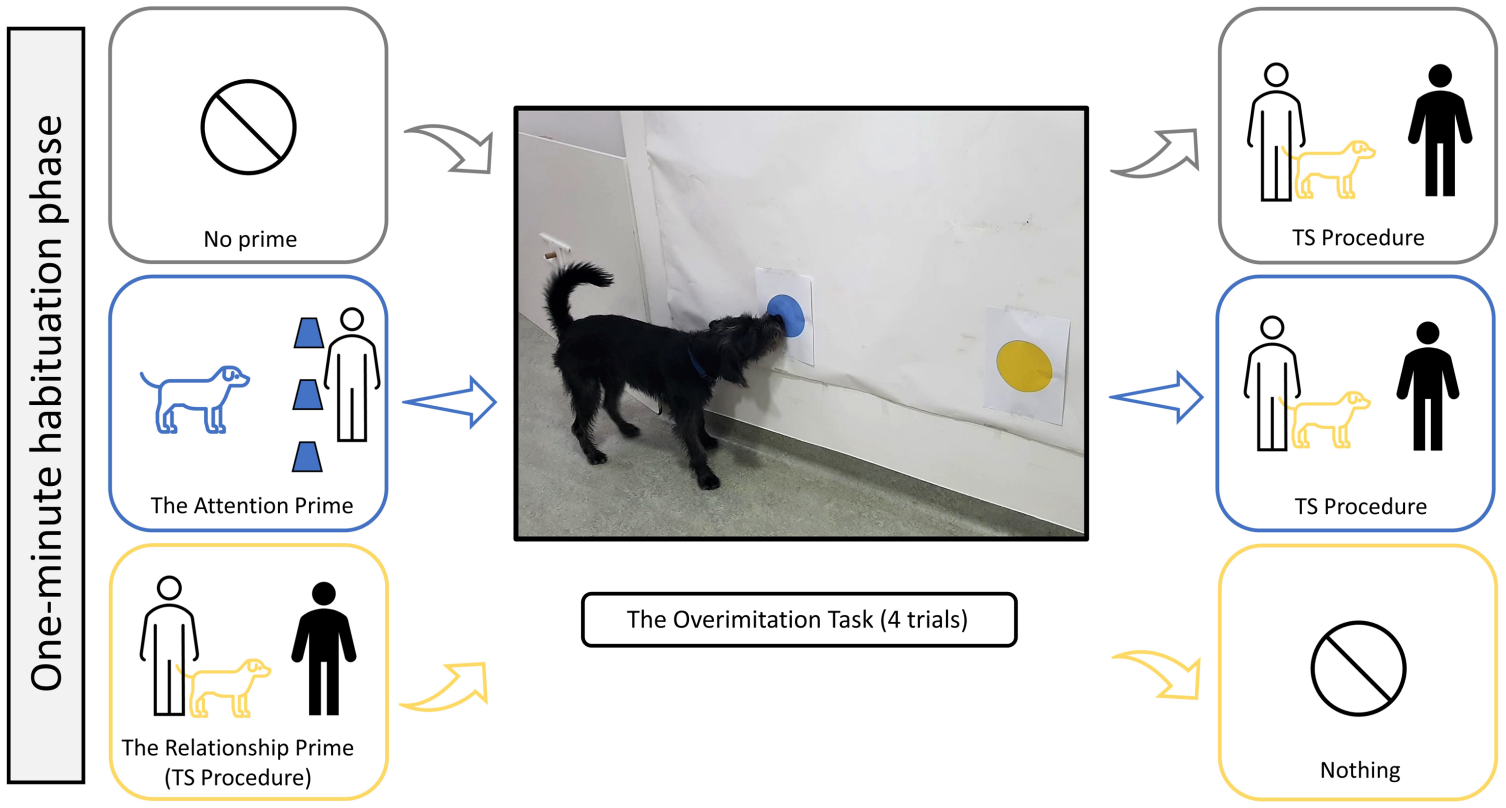
The order of events for the three priming conditions: no prime (gray) the attention prime (blue), and the relationship prime (yellow). In the middle is the overimitation task, with Filou (*male*) touching the blue dot of the irrelevant action, and to his left is the sliding door of the relevant action which hides a piece of sausage.

To test the effect of priming on a dog’s tendency to copy their caregiver’s actions, we assigned dogs to one of three conditions: (1) *the attention prime*, where dogs experienced a cup-game task with their caregiver prior to Huber et al.’s (2018) overimitation task, (2) *the relationship prime*, where dogs experienced the TS procedure prior to the overimitation task, and (3) *no prime (control)*, where dogs did not experience any priming. These three priming conditions were a between-subject factor, while the overimitation task had four trials as a within-subject factor, resulting in a mixed-design experiment. A video example of the primes and an overimitation trial is provided in the Supplementary material. A 1-min habituation phase occurred before the testing began, which allowed each dog to explore the testing room off-leash in the presence of both their caregiver and the experimenter. All dogs experienced the TS procedure to obtain a proxy measure of their attachment to the caregiver. This procedure was given either before or after the overimitation task, depending on the dog’s assigned condition (Figure 1). Following the procedure of Huber et al. (2018), dogs had a short break of around 3 min between the prime and the overimitation task, which also allowed the caregiver to watch the overimitation-task’s demonstration video and then practice out-of-sight. Face-masks were worn throughout the whole session due to COVID-19 regulations.

Once they arrived at the Clever Dog Lab, caregivers were greeted by the experimenter who then asked them to sign consent forms and complete a short canine-personality questionnaire (MCPQ-R; Ley et al., 2009). The caregivers were also verbally debriefed about the experimental procedure and told that they could withdraw at any point during the session. Testing then took place in a testing room (6.0 × 3.3 m) at the Clever Dog Lab (the same room as in Huber et al., 2018, 2020, 2022) with three video cameras recording for behavioral analysis.

#### 2.3.1. The overimitation task

To measure copying accuracy, we replicated the overimitation task from Huber et al. (2018), with the difference of including four trials for each dog instead of only one. The purpose of these trials was to observe any learning effects on copying behavior and compare them to Johnston et al.’s (2017) study, which used a traditional puzzlebox task. Additionally, the first trial may be the most influenced from a preceding prime because it is the closest trial to the priming event.

The overimitation task’s goal was to obtain a piece of sausage from a food chamber mounted on a wall-like wooden panel. Dogs had a 1-min chance to complete the task after observing their caregiver demonstrate an action-sequence, which contained both relevant and irrelevant actions toward this goal (Figure 1). There were two wall-based actions 130 cm away from each other, and at least 200 cm away from the subject, who was on the other side of the room. The goal-relevant action was to open a rectangular sliding door (10 × 9 cm) by pushing its handle (4 cm long and 2 cm diameter) with the nose (either left or right), gaining access to the food chamber (6 × 7 cm). The sliding door and food chamber were mounted in the center (50 cm from the floor to the handle) of a large wooden panel (150 × 100 cm). The goal-irrelevant action was to use the nose to touch at least one of two separate dots (15 cm in diameter) in the center of fresh A4 sheets of paper, which were stuck on the wall (around 45 cm from ground to the A4 sheet and 54 cm between the two A4 sheets) to the right of the wooden panel. These dots were newly printed for each dog in order to avoid scent-based interest from the previous dog/caregiver. Likewise, the sliding door and the test wall with the dots were wiped down with disinfectant between each dog. The dots and the wooden panel of the overimitation task were present in the room throughout the session. When not in use, the wooden panel had an empty food chamber and a locked sliding door. Dogs showed no interest in the dots during habituation and priming phases. The overimitation task’s design meant that the two actions were spatially distinct and disconnected from one another – providing the dogs with a clearer observation and understanding of action-relevancy toward the goal. This is in contrast to puzzlebox tasks, where both actions are performed on the same object. A small interactive puzzlebox could have led to accidental touches or misunderstandings as dogs have often trouble focusing on objects close to their snout, while the overimitation task’s spatial distance allowed for a higher degree of visual clarity.

To begin the overimitation task, the caregiver watched an instructional video for their task demonstration. Caregivers were asked to practice and perfect the task’s action-sequence before entering the testing room with the dog. When ready, she/he stood to the left of a chair where the experimenter was sitting and holding the dog’s leash, facing toward the test wall within the testing room. With the dog sat calmly between the caregiver and experimenter, the caregiver gave the dog some kibble to gain its attention and began the task demonstration. The caregiver first approached the wall with the dots and positioned her/him-self on her/his hands and knees in a dog-like manner – to ensure that the actions observed by the dog were anatomically suitable for copying. First, the caregiver made eye contact with the dog before touching the (left) blue dot with her/his nose, and repeated eye contact before touching the (right) yellow dot. Then, the caregiver moved toward the relevant action on the far left and, once again, looked at the dog before opening the sliding door leftward with her/his nose. She/he removed the treat from the chamber to show it to the dog before returning it. Eye contact during the demonstration was meant to establish ostensive-intentional actions and to ensure that the dog was indeed watching their caregiver. The caregiver lastly returned to the starting position (to the left of the dog) and was instructed to stand still and put her/his hands behind her/his back. To begin the trial, the dog was unleashed and released with a command from the caregiver (who was told not to interact with the dog during the trial). Each dog had a 1-min unaided opportunity to respond and solve the task before the room was reset for the next trial’s demonstration.

Dog behaviors of interest during the overimitation task included: irrelevant action copying, relevant action copying, approach behavior, and whether the irrelevant action was copied before or after obtaining the treat (the task’s goal). For further definition of each behavior, see Table 1.

**Table 1:** The list of behaviors coded from the video recordings with their scoring and descriptions.

Coded behavior	Score	Behavior description
*Irrelevant action copying*	0–3	Scored as 0 if the dog did not nose-touch any dots, 1 if the dog nose-touched one dot, 2 if the dog nose-touched two dots and 3 if the dog nose-touched two dots in the same order as the caregiver’s demonstration, during each 1-min trial
*Relevant action copying*	0–3	Scored as 0 if the dog did not nose-touch the sliding door, 1 if the dog nose-touched the sliding door, 2 if the dog opened the sliding door with their nose in any direction and ate the sausage, and 3 if the dog opened the door with their nose in the same direction as the caregiver’s demonstration, and ate the sausage, during each 1-min trial
*Approach dots*	Yes or no	Scored as yes if the dog positioned directly in front of the wall with the dots for at least one second, and scored as no if not, during each 1-min trial
*Approach food chamber*	Yes or no	Scored as yes if the dog positioned directly in front of the wooden panel for at least one second, and scored as no if not, during each 1-min trial
*Timing of irrelevant action*	Before or after	If the dog scored a 2 or higher for the relevant action copying and a 1 or higher for the irrelevant action during a 1-min trial, the timing of dot touching was scored as either before or after the dog opened the sliding door and ate the sausage
*Distance*	−50 to 200 cm	The distance from the caregiver (0 cm) where the dog spends the longest time during the TS procedure

#### 2.3.2. Attention priming

Dogs in the attention prime condition experienced a food-based cup-game (used in Huber et al., 2018) prior to the overimitation task. In the current study, performance was not measured, and the task was simply used like a warm-up to prime dogs to pay attention to their caregivers.

The caregiver watched an instructional video of the cup-game before entering the room with the experimenter, who was holding the dog on a leash. The experimenter sat in a chair ~2.5 meters away from three cups placed upside-down on the floor. The dog sat in front of the experimenter facing their caregiver, while the caregiver knelt behind the three cups to begin the game. The caregiver called the dog’s name and held up a piece of kibble, then placed it under the middle cup. She/he then placed her/his hands on her/his knees, looked at her/his dog before giving a release command. The dog was unleashed and allowed to choose a cup by sniffing it. The first cup sniffed was registered as the dog’s choice, and if correct, the dog was given the treat under the cup before returning to the starting position. If incorrect, the dog returned without any reward. This sequence was repeated six times, with the treat being hidden in the middle cup three times, then in the left (caregiver’s perspective), the right, and once again in the middle. At the end, the caregiver reclaimed her/his dog and left the testing room, while the experimenter then removed the cups to prepare for the overimitation task. The cup-game took ~3 min to complete.

#### 2.3.3. Relationship priming

All dogs experienced the Threatening Stranger (TS) procedure (similar to Vas et al., 2005) to assess each dog’s attachment to their caregiver, but only dogs in the relationship prime condition experienced the procedure *prior* to the overimitation task for priming the attachment-system.

The TS procedure took place in the testing room and required the caregiver to sit on a chair next to her/his dog on a two-meter leash. The caregiver was instructed to not interact with the dog and to remain neutral during the approach. The stranger was always a female researcher of the Clever Dog Lab who wore a long black raincoat with a facemask to standardize appearance for all the dogs (as in Solomon et al., 2019). She entered the room approximately four and a half meters away from the dog-caregiver pair and faced the dog to establish and maintain eye contact (an essential feature to appear threatening, Vas et al., 2005). She then took a half-meter step every 4 s to slowly approach the dog. Once she finally reached the dog, or if the dog showed signs of distress (i.e., growling), she would remove her hood and bend over to offer some food and a friendly greeting – this was to avoid any continued distress that might compromise the dog’s welfare. There were markings on the floor to indicate the half-meter steps and the distance for video coding. Overall, the procedure lasted around 1 min.

Whether or not dogs choose to hide behind their caregiver can be an indicator of the safe-haven effect of the dog-caregiver relationship. By hiding, dogs would actively be seeking proximity to (and protection from) their caregiver in the event of a threat (Gácsi et al., 2013). Seeking proximity in a social threat also has correlations with caregiver warmth and quality in their relationship (Cimarelli et al., 2016). Therefore, the distance held between the caregiver and the dog during the TS was used as a proxy measure of the dog-caregiver relationship’s safe-haven effect. There was a maximum score of 200 cm for the leash limit, and a minimum score of −50 cm if the dog maneuvered behind their caregiver (i.e., to hide). Distance was not recorded for six dogs who had caregiver-leash interference during the TS procedure, such as pulling back.

### 2.4. Behavioral coding and data analysis

#### 2.4.1. Behavioral coding

The video recordings each session comprised of three camera angles to allow precise detailed coding of the dog’s actions and behaviors. In particular, we had a close-up angle from above the dots to clarify whether the dog sniffed or touched the dot with its nose. These recordings were uploaded to Loopy (Loopbio, Vienna, Austria) for coding the following behaviors: *irrelevant action copying, relevant action copying, approach dots, approach food chamber, timing of irrelevant action,* and *distance*. All of the coded behaviors and their scorings are detailed in Table 1.

Personality ratings were summed and calculated as percentages from the MCPQ-R (Ley et al., 2009) for the following five variables; *extraversion, motivation, training focus, amicability,* and *neuroticism*. Each caregiver rated 26 adjectives between zero and six depending on how well the word described their dog (one being “really does not describe my dog” and six being “really describes my dog”). For example, *extraversion* contained words such as “energetic” and “excitable” (translated to German with a native-speaker). These scores were summed and converted to percentages by LM.

Additionally, dogs who overimitated (copied at least the touching of one dot) in their session were labeled as *overimitators*, while those who did not were labeled *non-overimitators*. This was to analyze their differences in *distance* and personality facets.

All 58 videos were coded by the experimenter (LM), while 12 (20%) of the videos were coded for reliability by an external coder who was naïve to the aims and hypotheses of the study. The agreement between the two coders was high for all the variables (Cronbach’s alpha >0.95).

#### 2.4.2. Data analysis

To estimate the effects that priming had on *relevant* and *irrelevant-action* copying accuracy (proportional odds response variables) we fitted two ordinal mixed models in R (Version 4.2.0; R Core Team, 2022) using the clmm function of the package “ordinal” (Version 2019.12-10; Christensen, 2018). Into the ordinal mixed models we included *priming*, *trial*, and their interaction as key test predictors, as well as *age* and *age squared* as control predictors. To account for repeated observations of the same individual as well as to avoid pseudo-replication we included the random intercept effects of *subject*. We additionally included the slope of trial within subject to avoid overconfident models and to keep the Type-I error rate at the nominal level of 0.05 (Schielzeth and Forstmeier, 2009; Barr et al., 2013). *Age squared* was chosen as there was a mild (non-significant) effect of young and old dogs having better copying scores in Huber et al. (2020). By z-transforming *trial* and *age squared* to a mean of zero and a standard deviation of one, they became standardized and more comparable.

Prior to fitting the full models, covariates *trial* and *age* were z-transformed to ease model convergence and achieve easier interpretable model coefficients (Schielzeth, 2010). After fitting the model we confirmed that model assumptions of proportional odds were not violated by dichotomizing the copying behavior as at least 1, at least 2 and at least 3, fitting logistic models with the obtained response variables, and inspecting the the model estimates. These varied only a little, suggesting the assumption was not strongly violated. We also verified absence of multicollinearity by calculating the Variance Inflation Factor (VIF) for a corresponding linear mixed model using the R package “car” (Version 3.0-12; Fox and Weisberg, 2018), which revealed that multicollinearity was not an issue (max VIF: 1.01). We assessed model stability with respect to the model estimates by comparing the estimates from the model (including all the data) with estimates obtained from models in which individuals were excluded one at a time (Nieuwenhuis et al., 2012). This revealed that the model is of good stability. The 95% confidence intervals for the model estimates and fitted values were calculated by applying the function “bootMer” of the package “lme4”, applying 1,000 parametric bootstraps. We compared the full model with a null model comprising only the threshold parameters and the fixed effect of age and age squared (but lacking the effects of priming condition and trial number) to avoid ‘cryptic multiple testing’ (Forstmeier and Schielzeth, 2011). The full ordinal mixed models are listed below. We tested the significance of individual fixed effects by dropping them from the full model starting with interaction and comparing the resulting model with the full model. If the interaction between priming condition and trial was non-significant, it was dropped from the full model to examine the reduced model (containing only the main effects).

Full ordinal mixed models:

relevant-action copying scores ~ priming condition * z.trial + z.age + I(z.age^2) + (1 + z.trial | Subject)
irrelevant-action copying scores ~ priming condition * z.trial + z.age + I(z.age^2) + (1 + z.trial | Subject)

Then, to examine whether priming, trial or age had an effect on the number of dogs who engaged in *relevant* and *irrelevant action* copying (copied/did not copy), two binomial mixed models were fitted in a similar way to the ordinal mixed models and their predictors (*priming*, *z*-*trial*, and *z-age squared*, with *subject*) in R, using the glmer function in the package “lme4.” For both types of actions, dogs in each trial were classified as *yes* for a copying score of at least one, or *no* for a copying score of zero. Similarly, two binomial mixed models were fitted with these predictors in R for the *approach dots* and *approach food chamber* response variables, also scored as *yes* or *no.* We also tested model stability with a full-null model comparison, and dropped the interaction between priming condition and trial if it was non-significant to create a reduced model (with only the main effects). The binomial mixed models are shown below.

Full binomial mixed models:

copied relevant actions yes/no ~ priming condition * z.trial + z.age + I(z.age^2) + (1 + z.trial | Subject)
copied irrelevant actions yes/no ~ priming condition * z.trial + z.age + I(z.age^2) + (1 + z.trial | Subject)
approach food chamber yes/no ~ priming condition * z.trial + z.age + I(z.age^2) + (1 + z.trial | Subject)
approach dots yes/no ~ priming condition * z.trial + z.age + I(z.age^2) + (1 + z.trial | Subject)

To examine *distance* between the caregiver and the dog during the TS procedure, we analyzed whether the average *distance* differed between dogs who overimitated and those who did not. We conducted a simple Welch’s *T*-test in R. Similarly, using the personality ratings from the MCPQ-R (Ley et al., 2009), we ran five two-sample *t*-tests with Bonferroni correction to compare overimitator and non-overimitator means for each personality facet (*extraversion, motivation, training focus, amicability* and *neuroticism*).

Finally, to examine the proportion of trials in which dogs copied the irrelevant action *before* (versus *after*) achieving the goal of the overimitation task, an exact binomial test was performed in R.

## 3. Results

**Figure 2:**
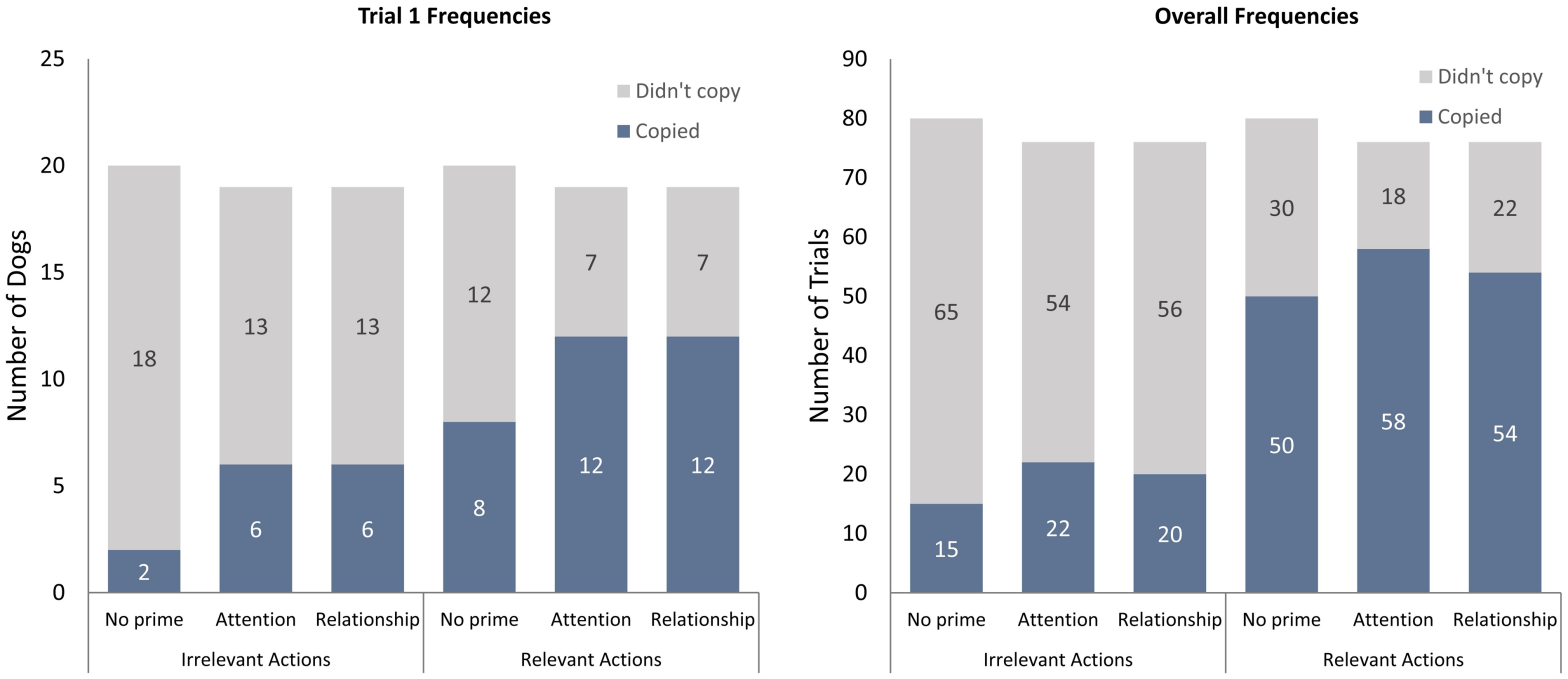
The copying frequencies for the three conditions: no prime (*N* = 20), attention prime (*N* = 19), and relationship prime (*N* = 19). The number of dogs who copied and did not copy irrelevant and relevant actions in their trials are depicted separately for trial 1 and overall (trial 1,2,3,4).

In total, 32/58 (55%) individual dogs in this study were classified as *overimitators* for copying their caregiver’s irrelevant action at least once during their four trials, and 52/58 dogs (90%) scored at least one yes for *approach dots* during their four trials. Nineteen of the *overimitators* copied the irrelevant action repeatedly (in more than one trial). Regarding only the first trial, 39/58 (67%) dogs scored yes for *approach dots* and 14/58 (24%) scored a 1+ for *irrelevant action copying*. The priming conditions’ copying frequencies for the first trial, and the four trials overall, are displayed in Figure 2.

### 3.1. Priming and copying accuracy

**Figure 3:**
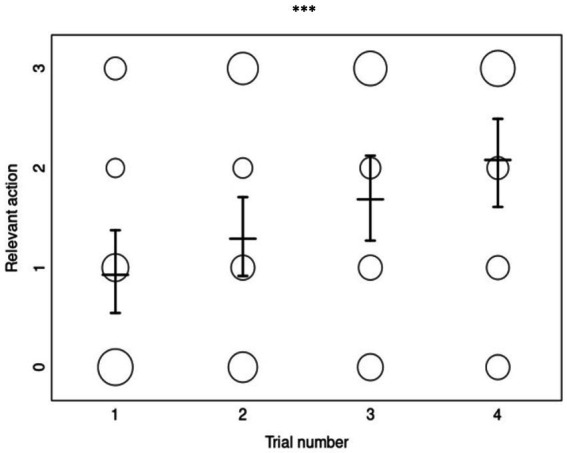
The overall relevant-action copying scores (0–3) as a function of trial number (*N* = 232). Each circle’s size represents the number of dogs who obtained the corresponding copying score, with the mean scores and error bars displayed for each trial number. “***” represents a *p*-value of <0.001 for the effect of trial in the ordinal regression analysis.

The full-null model comparison for the copying accuracy of *relevant actions* was clearly significant (*χ*^2^ = 22.997, df = 5, *p ≤* 0.001), confirming that our test predictors had an effect. Although, the interaction between priming condition and trial number was not significant (*χ*^2^ = 2.244, df = 2, *p =* 0.326) and was therefore removed from the model. The reduced model (containing only the main effects) revealed a significant effect of the number of trials, but not of priming or age (Table 2). The relevant-action copying accuracy of dogs did not differ significantly between priming conditions or by age, but generally increased per trial (Figure 3).

**Table 2:** The results of the reduced ordinal mixed model for the copying accuracy of relevant actions (*N* = 232), with Relationship Prime as the reference category for Condition. 0|1 represents the comparison between scores 0 and 1 of the relevant-action copying, 1|2 and 2|3 also represent the relevant-action copying scores.

Effect	Estimate	Std. Error	*Z*-value	Value of *p*
0|1	−2.012	0.947	−2.125	0.034
1|2	0.092	0.934	0.098	0.922
2|3	1.446	0.943	1.535	0.125
Attention Prime	0.585	1.059	0.553	0.580
No Prime (control)	−0.776	0.979	−0.793	0.428
Trial	**0.882**	**0.210**	**4.2**	**≤ 0.001**
Age	−0.157	0.459	−0.341	0.733
Age^2^	−0.061	0.426	−0.142	0.887

Values in bold have a significant *p*-value.

**Figure 4:**
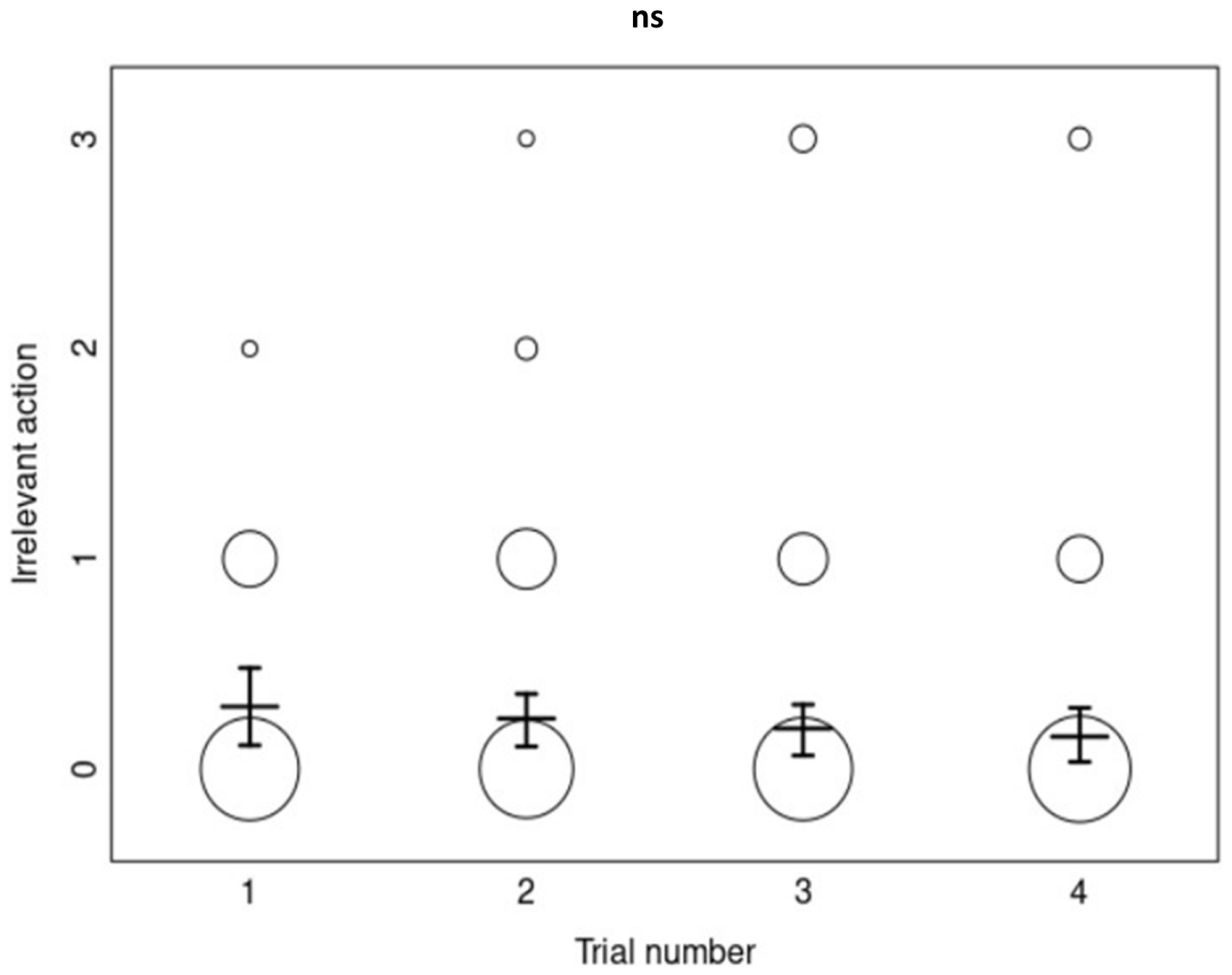
The overall irrelevant-action copying scores (0–3) as a function of trial number (*N* = 232). Each circle’s size represents the number of dogs who obtained the corresponding copying score, with the mean scores and error bars displayed for each trial number. “ns” represents a non-significant *p*-value of >0.05 for the effect of trial in the ordinal regression analysis.

In contrast, the full-null model comparison for *irrelevant actions* was not significant (*χ*^2^ = 3.767727, df = 5, *p =* 0.5833182), revealing no effects from the model’s predictors. The irrelevant-action copying accuracy of dogs did not differ significantly across priming conditions, trials, or age (Figure 4).

### 3.2. Priming and number of dogs copying

The full-null model comparison for the number of trials where dogs copied *relevant actions* was significant (*χ*^2^ = 15.766, df = 5, *p* = 0.007), and the non-significant interaction between priming condition and trial was removed (*χ*^2^ = 0.054, df = 2, *p =* 0.973). The reduced model showed a significant effect of trial, but not priming or age (Table 3). As with the copying accuracy scores, the number of dogs copying relevant actions increased per trial but did not differ between priming conditions or by age.

**Table 3:** The results of the reduced binomial model for whether or not dogs copied relevant actions (*N* = 232), with Relationship Prime as the reference category for Condition.

Effect	Estimate	Std. Error	*Z*-value	Value of *p*
(Intercept)	**26.387**	**4.917**	**5.366**	**≤ 0.001**
Attention Prime	−0.216	3.496	−0.062	0.951
No Prime (control)	−0.840	3.271	−0.257	0.797
Trial	**11.513**	**2.236**	**5.150**	**≤ 0.001**
Age	−0.399	1.490	−0.268	0.789
Age^2^	−0.256	1.373	−0.187	0.852

Values in bold have a significant *p*-value.

Again, similarly to copying accuracy scores, the full-null model comparison for the number of dogs copying *irrelevant actions* was not significant (*χ*^2^ = 3.09, df = 5, *p =* 0.686), therefore, there was no statistical difference across priming conditions, trials, or age.

### 3.3. Priming and approach behavior

The full-null model comparison for the number of trials where dogs approached the *food chamber* (relevant action) was not significant (*χ*^2^ = 1.744, df = 5, *p* = 0.883). There were no significant effects of priming, trial, or age on whether or not dogs approached the food chamber.

However, for the number of trials where dogs approached the *dots* (irrelevant action), the full-null model comparison was significant (*χ*^2^ = 14.08, df = 5, *p* = 0.015). After removing the non-significant interaction (*χ*^2^ = 1.777, df = 2, *p =* 0.411), there was a significant effect of trial and age, but not of condition in the reduced model (Table 4). Dogs approached the dots less per trial and less by age, but not depending on priming.

**Table 4:** The results of the reduced binomial model for whether or not dogs approached the dots (*N* = 232), with Relationship Prime as the reference category for Condition.

Effect	Estimate	Std. Error	*Z*-value	Value of *p*
(Intercept)	0.450	0.370	1.217	0.224
Attention Prime	−0.469	0.482	−0.973	0.330
No Prime (control)	0.008	0.480	0.017	0.987
Trial	**−0.508**	**0.160**	**−3.182**	**≤ 0.001**
Age	**−0.632**	**0.231**	**−2.731**	**0.006**
Age^2^	0.108	0.195	0.554	0.580

Values in bold have a significant *p*-value.

### 3.4. Safe-haven effect of attachment and overimitation

Of the dogs who were scored for *distance* during the TS procedure, 33/52 (64%) approached the stranger during the procedure (scored 50 cm or above), and all dogs accepted the food from the stranger at the end of the procedure once the threat was terminated. The mean distance between the caregiver and the dog for those who overimitated was 87 cm (*N* = 29, SD = 88.2 cm) and 81 cm (*N* = 23, SD = 89.1 cm) for those who did not. This difference was not statistically significant (Welch *t*-test: *t*(47.5) = 0.239, *p* = 0.812). Dogs who were overimitators did not seek proximity to their caregiver any more or less than dogs who were non-overimitators.

### 3.5. Timing of the irrelevant action in relation to the goal

Of trials where dogs both overimitated and reached the goal of obtaining the sausage, there was a much larger proportion of dogs copying the irrelevant action after (85%) than before (15%) achieving the goal, when compared to a 50:50 chance (*N* = 34, Exact binomial test: *p* ≤ 0.001).

### 3.6. Personality and overimitation (MCPQ-R)

**Figure 5:**
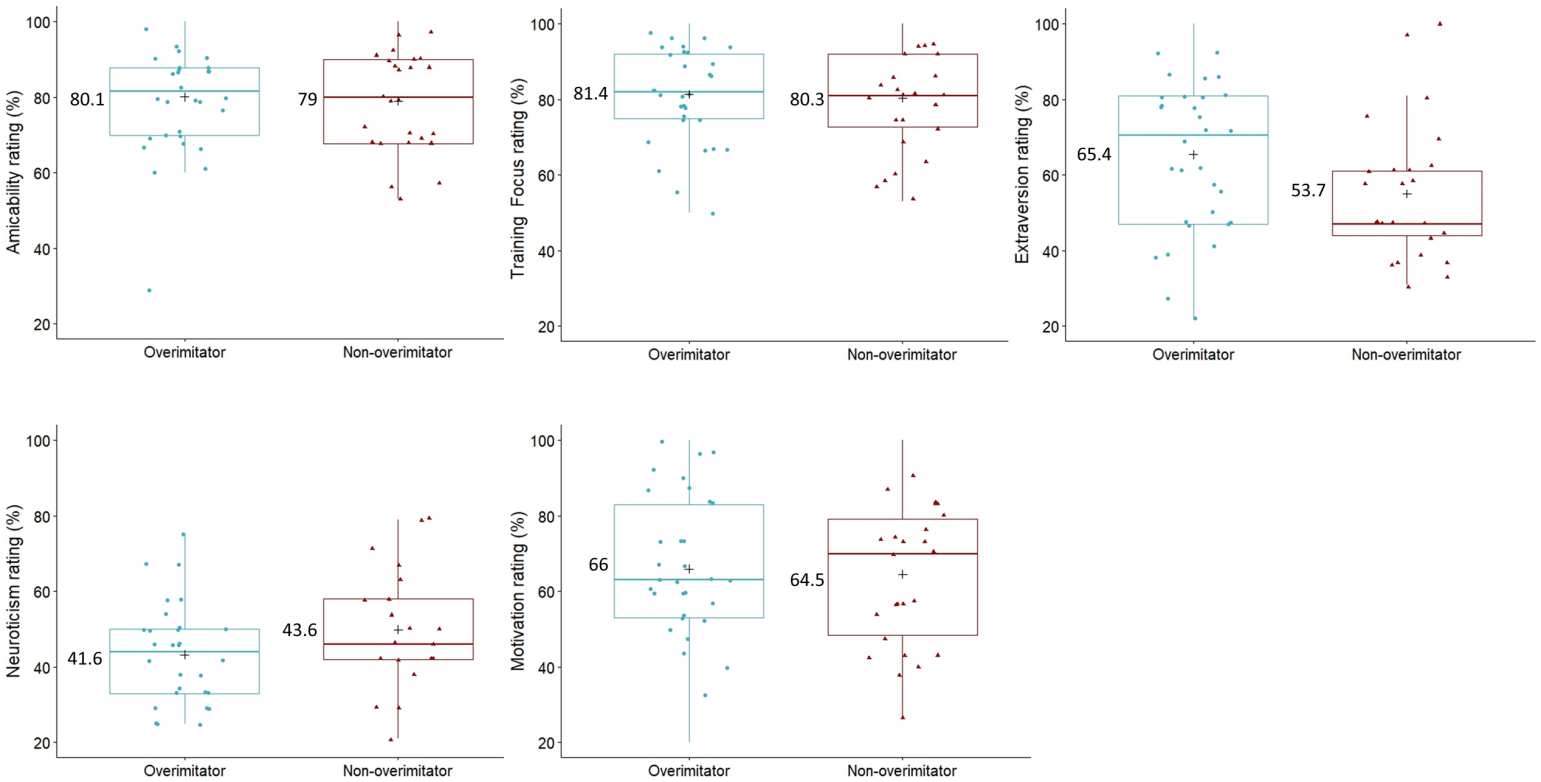
The boxplots of the caregivers personality ratings (% from the MCPQ-R; Ley et al., 2009) of their dogs (*N* = 58) for amicability, training focus, extraversion, neuroticism, and motivation. Dogs are grouped by whether they were labeled as an overimitator (*N* = 32) or not (*N* = 26), and the personality means (“+”) and mean values are displayed. Two sample *t*-test with Bonferroni correction revealed no significant differences between these groups for any personality facet.

After Bonferroni correction, there were no significant differences between the personality ratings (from the MCPQ-R; Ley et al., 2009) of dogs who overimitated and dogs who did not in terms of *extraversion* (Two sample *t*-test: *t*(56) = −2.245, *p* = 0.145), *motivation* (Two sample *t*-test: *t*(56) = −0.281, *p* = 1), *training focus* (Two sample *t*-test: *t*(56) = −0.294, *p* = 1), *amicability* (Two sample *t*-test: *t*(56) = −0.304, *p* = 1), and *neuroticism* (Two sample *t*-test: *t*(56) = 0.446, *p* = 1). Personality ratings and means for overimitators and non-overimitators are illustrated in Figure 5.

## 4. Discussion

The results of this study did not support our main hypothesis, that, like children, a dog’s motivation to imitate irrelevant or relevant actions can be influenced by priming. Even though there were less dogs copying these types of actions in the no-prime condition, the effects of the attention and relationship priming were not strong enough for statistical significance. We also predicted that overimitating dogs would show more safe-haven behavior during the TS procedure, however, they scored similarly to non-overimitating dogs, that is, they maintained a similar distance away from their caregiver when approached by the stranger. Further, our results showed that 85% of dogs overimitated their caregiver *after* already achieving the task’s goal (when dogs were both successful and overimitated in a trial). Overall, our results support the previous evidence of overimitation in dogs (Huber et al., 2018, 2020, 2022), since 55% of our dogs copied irrelevant actions at least once, and 67% of dogs approached the site of the irrelevant action in the first trial.

The selected primes may have been ineffective for a few reasons. One reason could be the ceiling effect of relationship-quality for our sample of dogs, as most dogs seemed to be securely attached to their caregivers. During recruitment, we asked that dogs were not aggressive toward strangers for safety, and volunteering caregivers tend to have healthy relationships with their dogs. Also, at the end of the TS procedure, all of our dogs were comfortable interacting with and taking food from the stranger. This behavior suggests that our dogs were comfortable to explore and utilize the secure-base from their caregiver’s presence, which is evidence of attachment-security (Solomon et al., 2019).

Another reason for the weakening of the relationship prime in particular could be due to the stranger’s greeting in the TS procedure. The greeting was to ensure that no stressful feelings persisted for our dogs, a practice which Over and Carpenter (2009) also followed with children by showing them a positive video clip after an ostracism prime. But such reassurance may have cost in terms of priming strength. Our greeting made the stranger less threatening and may have deactivated the attachment-system. It is also possible that the COVID-19 mask precaution has accustomed dogs to seeing only the eyes of strangers, making our masked stranger less threatening than, for example, the masked stranger in Solomon et al. (2019). For these reasons, the TS procedure was too mild to prime the attachment-system and assess individual differences. An alternative way to prime the dog-caregiver relationship without these problems could be to have a secure and neutral prime, such as a play or ignore session with the dog’s caregiver. This way, there would be no need to de-stress the dogs after priming, since positive feelings of the dog-caregiver relationship would be targeted.

The attention prime yielded the most imitated actions out of the three conditions, yet had no statistically significant effect on irrelevant or relevant-action copying. It is possible that any effect from the attention prime may not have reached statistical significance due to rather small the sample size (19 dogs in this group). Since priming consists of a subtle manipulation of mood or motivation, studies which use this method may wish to increase their sample or have greater contrast in their conditions. For example, we compared two relationship-based primes with a no-prime, where both primes included food rewards. The food reward may have equalized motivation for the relevant action between the attention and relationship prime, resulting in too small contrasts for any statistical relevance. In the future, it may be better to contrast positive and negative-feeling primes in relation to food and/or the caregiver. It would also be advantageous to find primes where the timing can be similar, as our relationship prime lasted 1 min while the attention prime lasted around three.

Regardless of priming, dogs copied relevant actions more often and more faithfully in subsequent trials, but not irrelevant actions. This means that not only did dogs become more motivated to get the sausage after observing multiple demonstrations and being rewarded for relevant-action copying, but they also copied the exact *direction* in which their caregiver opened the sliding door. Johnston et al. (2017) found that dogs and dingoes also became more successful at solving a puzzlebox after watching a demonstration for each trial. Although there was only one way to solve this puzzlebox (by opening a lid). Our finding supports those of Miller et al.’s (2009) study, which showed that dogs copied the same direction in which another dog or human demonstrator slid a screen to reveal food. Imitation requires one to copy the same strategy alongside the goal that they observe, even when other strategies are available. Although our study’s demonstrations only included the leftward push, dogs had four trials to experience the door opening both ways. If they had a lateral preference, they would have maintained this instead of matching their caregiver. Laterality preferences in dogs also tend to be found for paw-use (Tomkins et al., 2010), and dogs in the overimitation task used their nose to open the sliding door. By increasing relevant-action copying accuracy over trials, these dogs have shown behavior suggestive of directional imitation (of their human caregiver).

Additionally, our dogs did *not* significantly reduce their overimitation after subsequent trials (which they did in Johnston et al., 2017). This is a noteworthy finding, because it challenges the possibility that overimitation is a by-product of exploration behavior. If dogs were simply exploring the dots out of curiosity, they would stop overimitating after they experienced that the dots were non-functional. Our results showed that for each trial less and less dogs *approached* the wall with the dots. Yet, for each trial, a similar number of dogs chose to *copy* their caregiver’s dot-touching. By distinguishing approach behavior from copying behavior across the four trials, we observed a behavioral difference that seemed missed in previous studies. For example, Johnston et al. (2017) claimed a decrease in overimitation over their four trials, but their dogs were only scored for when they moved the puzzlebox’s irrelevant handle, which could have easily been due to accidental touches or initial curiosity. Our results provide a form of support for the thesis that dogs are genuinely overimitating rather than merely exploring the novel context in which the irrelevant action occurs.

The plausibility that the overimitation task produced overimitation rather than exploration is further strengthened by the task controls of Huber et al. (2018, 2020). In the original study of the overimitation task, dogs were either given a demonstration of *only* the relevant action (sliding door), or *only* the irrelevant action (dot-touching). When dogs observed the dot-touching alone, none managed to open the sliding door to obtain the food reward. Also, only two dogs (out of a combined 30 dogs between Huber et al., 2018, 2020) touched one dot after observing the sliding door relevant-action alone. In our study’s no-prime control we also saw only two dogs touching at least one dot in the first trial (after their caregiver’s demonstration). As these numbers were so low, it might be that it is not only important for dogs to *see* their caregiver showing irrelevant actions for them to overimitate, but also for dogs to be in a mood of engagement with their caregiver after primes. However, the effect of priming on imitation in dogs needs further testing to explore our data trends and statistical results (which showed a lack of an effect of priming).

With our distinction between approaching and copying, we also found that younger dogs were more likely than older dogs to approach the test wall with the dots, although they were not more or less likely to overimitate (and priming conditions were balanced for age). This may be because younger dogs are less experienced and are still learning about their caregivers. They might be more curious than older dogs to explore what their caregiver is doing. However, we only had data on the age of our dogs, not how much time they had spent together with their caregiver (for example, some dogs were likely adopted). In the future, strength of the dog-caregiver bond could be estimated by time-spent-together to reveal more information about motivations behind imitation in relation to age.

The final finding from this study was that dogs mostly overimitated after (85%) rather than before (15%) reaching the task goal, even though they observed the opposite order of events in the demonstration. This keenness for the sausage-reward is likely explained by dogs’ high food motivation and low inhibition, and may have taken priority over any desire to overimitate. Particularly, the two primes rewarded dogs with some kibble during the events, which may have facilitated their desire to go straight to the relevant action (although there were dogs in the no-prime condition who also overimitated after the reward). The goal-relevant action was also the last one to be shown in the demonstrations, suggesting a recency effect from memory. However, these dogs still chose to overimitate after there was no longer a reward to be obtained. Human children have been shown to overimitate much more if they observe an irrelevant action being demonstrated *after* the relevant action of a task, which Taniguchi and Sanefuji (2021) suggest is because they infer each action to have separate goals – one instrumental and one conventional (a social/normative goal). It is possible that dogs apply separate goals to the dot-touching and the door-sliding actions in our study; one goal to obtain food, and one goal to please or “be like” their caregiver.

We did not find any statistically significant personality differences between dogs who did or did not overimitate, but overimitators were rated (on average) 12% higher for their *extraversion*. Dogs who are more excitable or active could be more inclined to overimitate (or even imitate) their caregivers. Those who own dogs of such a personality type may wish to consider practicing the Do-as-I-do training method with them. This involves actions such as moving objects, sitting in boxes, and jumping, all of which dogs were shown to copy from their caregivers (Huber et al., 2009; Fugazza and Miklósi, 2014). Do-as-I-do training resembles mimicry behavior, as the actions are copied singularly rather than in a sequence. But mimicry has been compared to overimitation behavior in the sense that both types of copying include *seemingly* meaningless actions – actions that actually contain social signals rather than physically rewarding goals (Marsh et al., 2019). The do-as-I-do method can take advantage of a dog’s desire to copy their caregiver’s “meaningless” actions, and can uncover new possibilities for caregivers to train behaviors to dogs that are traditionally complicated to teach.

### 4.1. Future directions and conclusion

Although priming attention and the dog-caregiver relationship did not statistically influence dogs’ overimitation (or relevant-action copying) in this study, that is not to say that priming had no effect at all. There was a numeric trend of non-primed dogs having poorer performance in the overimitation task (Huber et al., 2018), and priming has drawn positive results in the imitation literature of humans (Over and Carpenter, 2009; Watson-Jones et al., 2016; Hopkins and Branigan, 2020). It is thus possible that other types of primes can affect dogs’ motivation to copy their caregiver. Future researchers who are interested in the social motivations behind overimitation could explore how positive primes, such as playing, might boost copying tendencies. A correlation has been found between the mimicry of other dogs’ behaviors and the continuation of cooperative play (Howse et al., 2018). So, a dog engaging in a play session with their caregiver might effectively prime dogs to overimitate for entertainment, as a kind of game (for the entertainment hypothesis of overimitation, see Schleihauf and Hoehl, 2020). There seems to be much potential for priming methodology in dog behavioral studies, and researchers may wish to consider the potential benefits of priming on dog task-performance.

All in all, our study showed that dogs copied the irrelevant actions of their caregivers regardless of priming, as with previous studies. But, by including four overimitation-task trials we were able to find that dogs copied their caregiver’s relevant-action more and more, and that dogs neither decreased or increased their overimitation across trials. We could not effectively assess the dog-caregiver attachment in regards to overimitation, however, dogs frequently overimitated their caregiver after already being rewarded for copying the goal-relevant action. This finding further points toward the notion of a social goal for dogs when they copy irrelevant actions. Finally, although we did not find statistically significant results regarding personality, there may be some relation between ‘extraversion’ (in terms of activeness) and dog overimitation tendencies.

## Data availability statement

The original contributions presented in the study are included in the article/Supplementary material, further inquiries can be directed to the corresponding author.

## Ethics statement

The animal study was reviewed and approved by University of Veterinary Medicine Vienna. Written informed consent was obtained from the owners for the participation of their animals in this study. Written informed consent was obtained from the individual(s) for the publication of any potentially identifiable images or data included in this article.

## Author contributions

LM, as first author, contributed to the conduction of the study, data collection, analysis, and the production of the manuscript. LH, as senior author, provided supervisor advice and input during the entire process of this study. All authors contributed to the article and approved the submitted version.

## Funding

The Austrian Science Fund (FWF: W1262-B29, www.fwf.ac.at), funded this research at the Veterinary Medicine University of Vienna.

## Conflict of interest

The authors declare that the research was conducted in the absence of any commercial or financial relationships that could be construed as a potential conflict of interest.

## Publisher’s note

All claims expressed in this article are solely those of the authors and do not necessarily represent those of their affiliated organizations, or those of the publisher, the editors and the reviewers. Any product that may be evaluated in this article, or claim that may be made by its manufacturer, is not guaranteed or endorsed by the publisher.
