# Corrigendum: Socially priming dogs in an overimitation task

**DOI:** 10.3389/fpsyg.2024.1412428

**Published:** 2024-04-29

**Authors:** Louise Mackie, Ludwig Huber

**Affiliations:** Comparative Cognition, Messerli Research Institute, University of Veterinary Medicine Vienna, Vienna, Austria

**Keywords:** overimitation, priming, dog behavior, social learning, comparative psychology

In the published article, there was an error in the caption for Figure 4 as published. The caption states “relevant-action copying” instead of “irrelevant-action copying” and “ < 0.05” instead of “>0.05”. The corrected Figure 4 caption is shown below.

“Figure 4. The overall irrelevant-action copying scores (0–3) as a function of trial number (*N* = 232). Each circle's size represents the number of dogs who obtained the corresponding copying score, with the mean scores and error bars displayed for each trial number. “ns” represents a non-significant *p*-value of >0.05 for the effect of trial in the ordinal regression analysis.”

The authors apologize for this error and state that this does not change the scientific conclusions of the article in any way. The original article has been updated.

